# Motivating factors and possible barriers to participation in digital prevention courses of two statutory health insurance funds in Germany: a qualitative interview study

**DOI:** 10.1186/s12889-026-28392-z

**Published:** 2026-07-07

**Authors:** Michelle Brodski, Trix Twelkmeyer, Dunja Bruch, Susann May, Felix Muehlensiepen, Frances Seifert, Sebastian Spethmann, Gerrit Fleige, Marc Lehnen

**Affiliations:** 1 revFLect Advisors GmbH, Joachim-Friedrich-Straße 37/38, Berlin, 10711 Germany; 2 Department of Cardiology, Angiology and Intensive Care Medicine, German Heart Center at Charité – Universitätsmedizin Berlin, corporate member of Freie Universität Berlin and Humboldt-Universität zu Berlin, Berlin, Germany; 3 Centre for Health Services Research, Brandenburg Medical School Theodor Fontane, Brandenburg, Rüdersdorf bei Berlin Germany; 4 Department of Cardiology, Angiology and Intensive Care Medicine, German Heart Center at Charité – Universitätsmedizin Berlin, corporate member of Freie Universität Berlin and Humboldt-Universität zu Berlin, Berlin, Germany; 5 DZHK (German Centre for Cardiovascular Research), Partner Site Berlin, Berlin, Germany; 6 Theodor Fontane Medical University of Brandenburg, Neuruppin, Germany

**Keywords:** Digital prevention, Digital health, Health promotion, Behavioral prevention, Statutory health insurance, Germany, Qualitative study

## Abstract

**Background:**

Digital prevention courses offered by statutory health insurance funds in Germany represent a low-threshold opportunity to support health-promoting behaviors through pre-recorded, video-based programs focusing on exercise, nutrition, or stress management. However, little is known about insured individuals’ motivations and barriers to participation. This study aimed to identify relevant motivating and obstructive factors for participation in digital prevention courses and to explore differences compared to on-site formats.

**Methods:**

We conducted semi-structured telephone interviews with 20 insured members of two large regional statutory health insurance funds in Germany (AOK North-East and AOK North-West) who had recently completed digital prevention courses. Participants were recruited via e-mail invitations sent by the health insurance funds following course participation. Five additional interviews were conducted with participants of on-site courses for contextual comparison. Interviews were conducted between February and December 2023, transcribed verbatim, and analyzed using qualitative content analysis with a deductive–inductive coding approach based on the study objectives and interview guide.

**Results:**

The sample included 20 participants (mean age 41 years, range 25–72; 19 women and 1 man) who had completed digital prevention courses. Participants emphasized temporal flexibility, home-based access, and low preparation effort as key facilitators of participation. Additional motivators included health-related goals, free access, practical content, reminders, and the possibility to repeat sessions. Reported barriers included a lack of discipline, reduced social interaction and commitment compared to on-site formats, and work- and family-related demands. The availability of recorded sessions was perceived as a major advantage that facilitated course completion. Participants also valued competent instructors, but called for clearer differentiation of difficulty levels, broader course options, continued access to materials, and follow-up opportunities. The contextual comparison showed that on-site course participants particularly valued the sense of community and the possibility of individual corrections by instructors.

**Conclusions:**

Digital prevention courses are well accepted and can promote participation due to their flexibility and accessibility. However, challenges such as reduced social interaction and the need for self-discipline limit their full potential. To enhance their effectiveness and reach, digital prevention programs should integrate more tailored content, structured follow-up offers, and interactive or hybrid elements that foster engagement and social connectedness.

**Trial registration:**

German Clinical Trial Register DRKS00029761; Registration date 27.07.2022; https://drks.de/search/de/trial/DRKS00029761.

**Supplementary Information:**

The online version contains supplementary material available at 10.1186/s12889-026-28392-z.

## Background

 Individual behavior-based preventive measures play an important role in promoting health-related behaviors and integrating them into daily life [1]. These measures target to influence lifestyle and personal choices, such as dietary patterns, physical activity, and stress management, to improve individual health behaviors and prevent widespread conditions like diabetes, cardiovascular diseases, or mental health disorders [2, 3]. The individual prevention measures may include various interventions, such as counseling services or group courses [1, 2].

In Germany, statutory health insurance providers support preventive courses for their insured members based on the legal framework defined in the Social Code Book V (Sozialgesetzbuch (SGB) V, § 20). While the exact reimbursement conditions may vary between providers, many statutory insurers cover most or all the course costs when participation requirements are fulfilled. Individual prevention courses typically address lifestyle-related topics such as physical exercise, nutrition, stress management, and substance use (e.g., smoking cessation, alcohol reduction) [2]. These programs represent an important component of the German prevention strategy, aiming to reduce health risks and strengthen individual health resources [1, 4]. Within this framework, prevention courses must meet defined quality standards to be subsidized by statutory health insurance funds. Quality assurance is organized through a centralized certification system. The Central Prevention Testing Center (Zentrale Prüfstelle Prävention, ZPP) evaluates whether course concepts and instructors comply with the quality criteria defined in the Prevention Guide [4] which operationalizes the legal requirements of § 20 SGB V. Courses that successfully pass this review receive certification that allows statutory health insurance funds to subsidize participation. According to the statutory health insurance prevention report [5], this certification process aims to ensure evidence-based quality standards in individual behavior-based prevention programs.

Despite this structured framework, the effectiveness of individual behavior-based prevention courses has been subject to ongoing debate. Critics argue that many interventions insufficiently incorporate insights from behavioral and social sciences and often rely on simplified assumptions about how information and knowledge translate into behavioral change [1]. In addition, the overall evidence base for prevention and health promotion interventions remains limited, particularly regarding long-term behavioral outcomes [6–8]. Existing studies generally report moderate effects of lifestyle-oriented prevention programs on participants’ behavior, while robust evidence on sustained behavioral change is scarce [6, 9, 10]. Assessing effectiveness is further complicated by the considerable heterogeneity in course content, duration, and methodological approaches used in evaluation studies. The most recent statutory health insurance prevention report also highlights existing evidence gaps and indicates that results from a comprehensive evaluation study of on-site prevention courses in the field of physical activity are expected to be published in 2026 [5]. Nevertheless, individual prevention courses are widely offered by the health insurance funds in Germany and remain an important part of the implementation of the national strategy to promote a healthy lifestyle and to prevent diseases.

Previous studies have shown that various individual-level factors influence the utilization of preventive health services. For example, sociodemographic characteristics such as age and gender play an important role: prevention courses are primarily used by women and middle-aged to older adults (approximately 50–70 years old) [1, 11, 12]. A medium to high level of education and socioeconomic status are also associated with increased participation in such courses. Alongside sociodemographic factors, health literacy, defined as the ability to access, understand, evaluate, and apply health information, also plays a crucial role in engagement with preventative health [13, 14]. Low health literacy is associated with poorer health outcomes and less effective use of health care services. For example, individuals with low health literacy are less likely to participate in prevention programs, including cancer screening or vaccination programs [15–19]. In addition, previous studies have shown that individuals who make use of prevention courses tend to have a positive attitude towards health and already exhibit a high level of health-promoting behavior (e.g., physical activity, healthy eating, and non-smoking) [1, 9]. To motivate individuals who generally exhibit lower levels of health-promoting behavior to participate in prevention courses, statutory health insurance providers implement various measures. These include, for example, promotional efforts via newsletters or social media platforms [9, 20].

Another approach to reach a broader segment of the population and to encourage participation in preventive programs is the provision of digital courses. Digital prevention courses received a huge boost, especially during the coronavirus pandemic, as it was no longer possible to attend on-site courses for precautionary reasons [21]. Digital prevention courses are characteristically more accessible, as they are associated with lower entry barriers and reduced preparation effort [22]. For example, there is no need to travel to and from a course location, which may facilitate participation for individuals with limited mobility, those living in rural areas, or people with time constraints [14, 23, 24]. In addition, participants are not exposed to direct social comparison within a group setting. According to the prevention report 2024 of the health insurance funds in Germany, information on on-site versus digital formats was available for 1.499.987 course participations in 2023. Of these, 78% were on-site participations (*n* = 1.173.824), whereas 22% were performed digitally (*n* = 326.163) [12]. This corresponds to approximately XX Indeed, health insurance providers successfully reached new target groups through the provision of digital prevention programs. Younger individuals were more likely to participate in digital courses compared to older adults, who tended to favor on-site formats. Specifically, 68% of participants in digital courses were between 20 and 49 years of age, whereas 63% of participants in on-site courses were over 50. Moreover, men, who are typically underrepresented in traditional prevention programs, were more effectively reached through digital offerings than through on-site courses. In on-site courses, only 19% of participants were male, while in digital courses this proportion increased to 28% [12].

However, despite the growing availability of digital health promotion and disease prevention services, their general uptake remains uneven. Research shows that the use and acceptance of digital health services depend on factors such as digital health literacy, perceived usefulness, trust, and sociodemographic characteristics [25, 26]. For the German context in particular, recent nationwide surveys indicate that digital health services are still not routinely used and that many individuals continue to prefer traditional, non-digital health services despite increasing digitalization of the health sector [27, 28]. This may partly reflect the comparatively broad availability of conventional healthcare services and prevention programs in Germany, including local sports clubs or community-based health promotion activities [27]. In addition, this preference may be related to comparatively low levels of digital health literacy in parts of the population [26]. Against this background, it remains important to better understand motivations and barriers related to digital prevention programs within the specific German healthcare context.

The present study therefore aimed to identify relevant motivating factors and possible barriers to participation in digital prevention courses of two local statutory health insurance funds in Germany. Specifically, we aimed to systematically identify individual, structural, and application-related factors that are associated with the use of digital preventive courses offered by the AOK North-East and AOK North-West. Both AOK funds belong to one of the largest statutory health insurance providers in Germany and insure a broad and socioeconomically diverse population across urban and rural regions. Although the present study focuses on participants of digital prevention courses offered by the two AOK funds, these courses are embedded within the broader framework of statutory health insurance–based prevention programs in Germany. Therefore, the findings are intended to provide insights into general patterns of use, motivations, and barriers related to digital prevention courses in the statutory health insurance context. To address these aims, we conducted a qualitative interview study with 20 insured individuals on their motivation to participate in the digital courses, the advantages and disadvantages of digital versus on-site measures, and possible barriers to their participation. Moreover, given that individuals with a positive attitude towards health and pre-existing health-promoting behavior are more likely to engage in preventive measures [1], this study also aimed to investigate how both past and current health behaviors relate to participation in digital prevention courses. To contextualize and deepen the findings, additional interviews were carried out with participants of on-site prevention courses, allowing for an exploratory comparison between digital and on-site formats. By gaining a deeper understanding of motivational factors and barriers, the results may support the further development of digital prevention programs and their alignment with the needs and preferences of insured individuals. In the long term, this knowledge could also contribute to reaching broader population groups and motivating them to participate in preventive courses.

The digital prevention courses examined in this study were offered by CyberFitness through the regional health insurance funds AOK North-East and the AOK North-West. Every person who is insured at these health insurance funds can participate at up to two digital or on-site prevention courses per year free of charge. To receive full reimbursement, participants must have attended at least 80% of the course sessions. The AOK funds actively encourage participation through their website and newsletters.

The courses cover various topics, including exercise (e.g., fasciae training, back-fitness), stress management (e.g., yoga, resilience, autogenic training), nutrition (e.g., healthy eating, weight management), and management of substance use (e.g., smoking cessation, alcohol reduction). Based on participants’ reports, the courses typically combined theoretical input with practical exercises. Theoretical components included, for example, information on physiological, behavioral, and psychological aspects of health-related behaviors, while practical components focused on the application of this knowledge in everyday life (e.g., guided exercises, cooking demonstrations, or coping strategies). However, the specific structure and content varied between courses. A more detailed overview of reported course characteristics, including illustrative examples based on participants’ accounts, is provided in the Supplementary material 1. Further information on course content and structure is available on the websites of the respective AOK health insurance funds (https://www.aok.de/pk/gesundheitskurse/).

The digital courses are pre-recorded and can be activated weekly; hence not all videos are available at the same time. Unlocking the next video is only possible once the previous video has been watched. Within the 8–10-week course period, the activated videos can be accessed flexibly, and repetitions are possible. The course format does not include live sessions, interactive elements, or direct contact with course instructors or other participants. After the course ends, the videos are no longer accessible, but relevant text material can be downloaded and saved on the computer.

## Method

### Study design

This study is part of the research project “Digital health preventive measures for arterial hypertension” (DiPaH), which aims to identify structural and individual factors influencing the use of digital prevention measures for arterial hypertension in Germany, with particular attention to regional differences, age-related factors, and digital health literacy [23]. While the overall project focuses on hypertension-related prevention, the present study examines general patterns of participation in digital prevention courses across different content areas. Specifically, the study focuses on digital prevention courses offered within the statutory health insurance system in Germany, using insured participants as a sampling frame. The aim is not to evaluate all prevention measures or to compare different providers, but rather to explore user perspectives on participation, motivations, and barriers in digital course formats. These courses typically address lifestyle areas (e.g., physical activity), which are known to influence blood pressure and reduce cardiovascular risk. The study is therefore relevant within the broader research context of digital prevention of arterial hypertension.

### Material

The interview guide was developed specifically for the study objectives and focused on motivation for participation, experienced barriers, and perceived advantages and disadvantages of digital prevention courses. The interview guide is provided in Appendix A. Its development was informed by existing literature on preventive health behavior and the use of digital health services, as well as the research questions of the DiPaH project [1, 9, 23]. The guide also included questions on sociodemographic characteristics (e.g., sex, age, educational level, residential area). Its development followed an iterative process within the research team, involving multiple rounds of discussion, feedback, and refinement to ensure clarity, comprehensibility, and alignment with the study aims. A formal pre-test of the interview guide was not conducted.

### Data collection

In the present study, semi-structured interviews were conducted with members of the AOK North-East and AOK North-West between February and December 2023. A purposive sampling strategy was used to recruit insured individuals who had recently completed a digital prevention course and were able to provide in-depth insights into their motivations, perceived barriers, and experiences with the course format. No specific quotas (e.g., for gender or age) were applied. The number of interviews was guided by the principle of thematic saturation, defined as the point at which additional interviews no longer yield new relevant themes. Methodological literature provides orientation regarding adequate sample sizes for qualitative interview studies. For example, it has been suggested that meaning saturation is often reached after approximately 16–24 interviews [29]. Similarly, around 16 interviews may be sufficient to identify the main themes in relatively homogeneous groups and focused research contexts [30]. Based on these considerations, a sample size of 20 interviews was considered appropriate to capture both thematic patterns and a sufficient depth of understanding within the study population of prevention course participants.

In line with the study focus on digital prevention, an additional, smaller on-site course subsample was included to provide contextual insights and enable exploratory comparison between formats. For this type of contextual and contrastive analysis, a limited number of interviews with on-site course participants is methodologically sufficient, as this group primarily served to situate and theoretically enrich the findings from the digital subsample [31].

Potential digital and on-site course participants were contacted via e-mail following their course participation and were invited to take part in the interview study. Only individuals who expressed interest were subsequently contacted by telephone, during which the study aims and procedures were explained and an interview appointment was scheduled. The interviews were conducted via telephone in German by two members of the research team (D.S. and M.B.), who were trained in qualitative research methods and had no prior relationship with the participants.

Participants were required to be at least 18 years old and to provide consent to participate in the study. All interviewees took part voluntarily and received detailed information about the study, its aims, procedures, and data protection measures before providing written informed consent for the use of their data. All interviews were audio-recorded with participants’ consent and were anonymized after transcription. Participants received 30€ as reimbursement.

### Data analysis

The digitally recorded interviews were transcribed verbatim. Qualitative content analysis was applied to the interview data in German [32] using MAXQDA Analytics Pro 2022, Release 22.1.0, Verbi GmbH (Berlin, Germany). Coding was conducted by E.B. and M.B. Main- and subcategories were developed and revised through iteration according to a deductive-inductive procedure. Initial categories were derived from the study objectives and the thematic structure of the interview guide [32]. During analysis, the coding framework was iteratively refined: additional subcategories were generated inductively from the data, and existing categories were adapted where the interview material suggested modifications [32]. Data and categories were discussed within the research group until no new themes could be identified and code saturation was achieved [29]. Representative quotes for each category were translated into English to present the findings (see Results). The study was conducted and reported in accordance with the COREQ (Consolidated Criteria for Reporting Qualitative Research) checklist [33]. The COREQ checklist is provided as Supplementary material 2, with additional information presented in Supplementary material 3.

## Results

A total of 20 interviews were conducted with participants of digital prevention courses. The interviews had a mean duration of 26 min (range 16–41 min). 19 participants were female, and one participant was male. Their mean age was 41.15 years (range 25–72) and most participants’ educational background was high (i.e., high school diploma) (see Table 1). The participants resided in different types of regions, categorized based on population size and reflecting an urban-rural continuum (see Table 1). When participants were asked how they became aware of the course, they reported various sources of information, including their own independent research, the AOK newsletter, the AOK website, direct contact with the AOK, and social media. Additionally, some participants indicated that they have previously taken part repeatedly in AOK prevention courses.

Additionally, five interviews were carried out with participants of on-site prevention courses. The interviews had a mean duration of 27.2 min (range 23–35 min). The mean age of the participants was 56.6 years (range 23–76), and all interviewees were female.

In total, 35 interested individuals were contacted by phone, of whom 25 completed the interview (20 digital and five on-site course participants), corresponding to a participation rate of 71.4%.

**Table 1:** Sample characteristics

ID	Age	Gender	Educational background	Residential area	Prevention course type	Prevention course subject
ID01	37	female	High school	Metropolis	Digital	Yoga
ID02	54	female	Not specified	Metropolis	Digital	Autogenic training
ID03	57	female	High school	Small city	Digital	Smoking cessation
ID04	34	male	Secondary school	Metropolis	Digital	Stress management
ID05	33	female	High school	Metropolis	Digital	Yoga
ID06	72	female	High school	Small town	Digital	Nutrition
ID07	46	female	Secondary school	Metropolis	Digital	Back-fitness
ID08	25	female	High school	Metropolis	Digital	Nutrition and vitality
ID09	47	female	High school	Medium-sized city	Digital	Back-fitness
ID10	26	female	Secondary school	Small city	Digital	Stress and Time management
ID11	46	female	High school	Medium-sized city	Digital	Yoga
ID12	28	female	High school	Metropolis	Digital	Losing weight
ID13	27	female	High school	Metropolis	Digital	Back-fitness
ID14	37	female	High school	Metropolis	Digital	Yoga
ID15	40	female	High school	Medium-sized city	Digital	Yoga
ID16	29	female	High school	Metropolis	Digital	Pilates
ID17	30	female	High school	Medium-sized city	Digital	Yoga
ID18	63	female	High school	Medium-sized city	Digital	Back-fitness
ID19	46	female	High school	Small town	Digital	Losing weight
ID20	46	female	High school	Small city	Digital	Nutrition and losing weight
ID21	37	female	Vocational School	Small town	On-site	Back-fitness
ID22	23	female	High school	Metropolis	On-site	Back-fitness
ID23	76	female	Vocational School	Not specified	On-site	Back-fitness
ID24	76	female	Secondary school	Small city	On-site	Back-fitness
ID25	71	female	Secondary school	Small city	On-site	Back-fitness

Educational Background: High school = 12–13 years of schooling, Vocational school = 12 years of schooling, Secondary school = 8–10 years of schooling. Residential area: Metropolis = from 1.000.000 residents, Large city = from 100.000 residents, Medium-sized city = from 20.000 residents, Small city = from 5.000 residents, Small town = under 5.000 residents

The results showed that various motivational and obstructive factors are associated with participation in digital prevention courses. Thereby four main themes emerged from the analysis:

1. Motivating and supporting factors for course participation.
2. Perceived challenges and barriers to participation.
3. Past and present health behavior.
4. Course evaluation and suggestions for future courses.

**Fig. 1:**
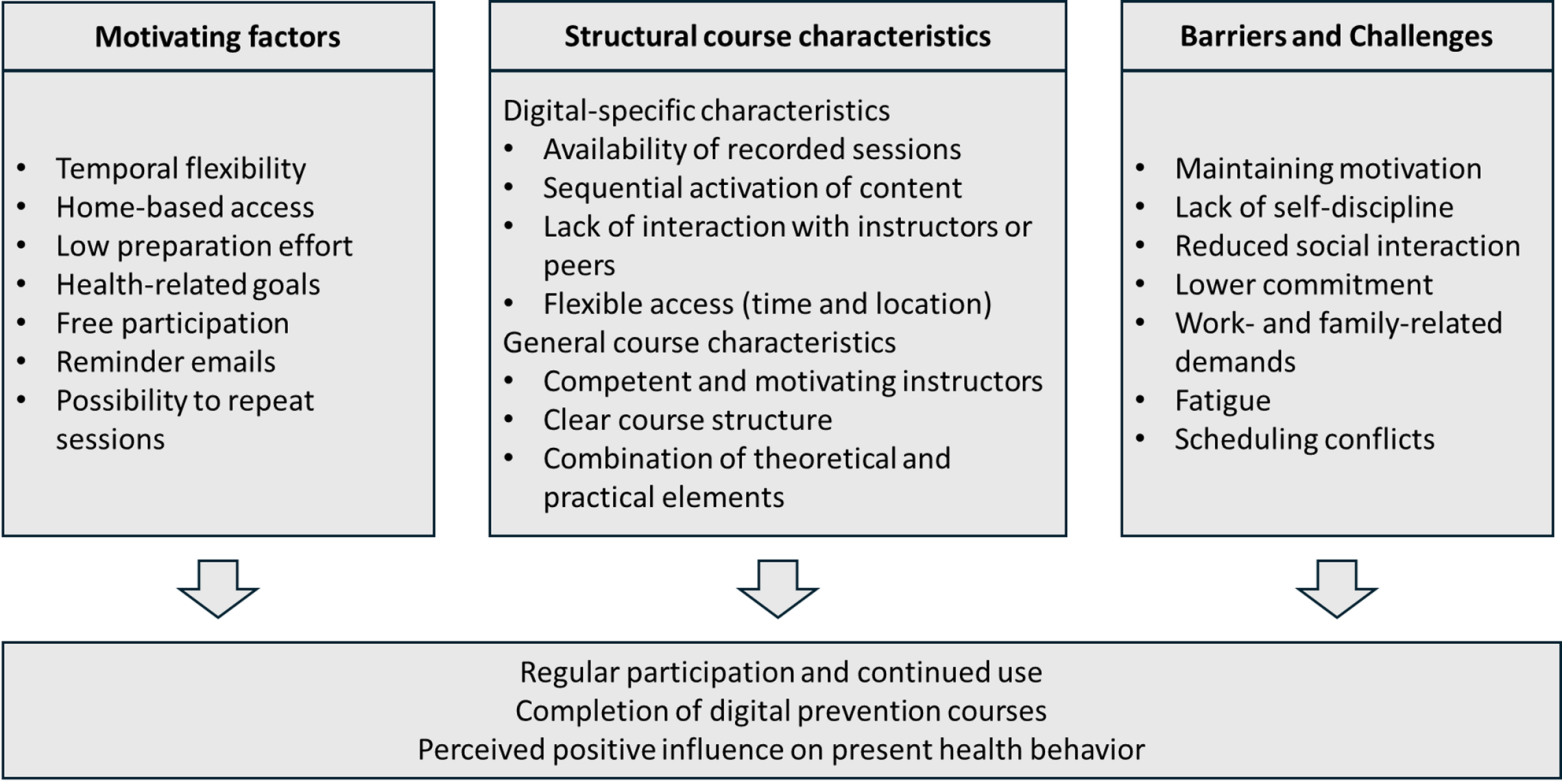
Factors associated with participation in digital prevention courses

Figure 1 provides an overview of how key factors identified across these themes are associated with the use of digital prevention courses. The coding framework, including main and subcategories, is provided in Appendix B.

### Motivating and supporting factors for course participation

Participants emphasized that flexible scheduling and a high degree of flexibility were key supporting factors contributing to their engagement in the digital courses. The absence of travel requirements and the resulting low preparation effort further facilitated participation, particularly for individuals with family responsibilities (e.g., caring for young children). Participants reported a range of health goals, including becoming more active, practicing self-care and relaxation, losing weight, adopting healthier eating habits, and addressing specific health concerns such as pain management, hypertension control, or smoking cessation. These goals served as central motivators for course participation. Free access to the courses was also identified as an important facilitating factor. In addition, participants were also motivated by the presentation of the course content and the increase in knowledge, particularly when it combined practical applicability with informative elements.

For example, participants valued helpful nutrition tips and easily implementable recipes, as well as non-lecturing formats in the smoking cessation course that combined practical advice with interesting psychological background information (see Supplementary material 1). Further supporting factors included the possibility to repeat sessions and exercises, as well as reminder emails that helped participants maintain engagement. Social encouragement from family or friends played only a minor role in digital course participation, as it was reported by only one participant. See Table 2 for corresponding subcategories and respective anchor quotes.

**Table 2:** Motivating and supporting factors for course participation: subcategories and anchor quotes

Subcategory	Anchor quote
Flexible scheduling and a high degree of flexibility	“Because of the fact that you could just access it anytime. (…) If I had fixed appointments, it might not have worked. I have three children and a dog, so unexpected appointments come up. But when there was such an appointment, then I did the course in the evening. So, it was actually very, very easy for me to stay engaged with it.” (ID03, Pos. 51)
Achievement of health goals	“Not, I would say, to become healthy, but rather, to stay healthy and flexible, especially. I think, in everyday life, one becomes less and less mobile. And Yoga and Pilates are really good for bringing in a bit more flexibility.” (ID01, Pos. 36)
Free access	“But I have to say, I simply looked at what was offered by the health insurance. Basically, within the things where you wouldn’t have to spend additional money. That was actually a main reason.” (ID12, Pos. 86)
Presentation of content and increase in knowledge	“So, in the quit-smoking course, it was really great that they didn’t just say with a wagging finger that smoking is bad, but they also addressed a bit the psychological reasons. You get some guidance on how to handle it if you crave a cigarette, how to get through it, and that it will pass after a few minutes. Things like that.” (ID03, Pos. 27–29)
Multiple viewing and repetition of exercises	“Then you could also rewatch the last week and repeat the other exercises. I found that very good. Especially, because you could do it several times a week.” (ID07, Pos. 56)

### Perceived challenges and barriers to participation

Participants encountered several challenges related to participation in digital courses. Maintaining motivation when completing the course independently at home was described as difficult compared to on-site formats. The digital format requires a higher degree of self-discipline, as the absence of fixed appointments and direct social interaction reduces the sense of commitment. The lack of group dynamics and personal exchange was therefore perceived as a key barrier to sustained engagement. In addition to these format-related challenges, participants also reported more general barriers to course participation. Work-related demands, fatigue, and scheduling conflicts (e.g., medical appointments, childcare responsibilities, or illness) sometimes interfered with participation.

At the same time, certain features of the digital format helped participants to compensate for these barriers. The availability of recorded sessions allowed participants to watch content at a later time (e.g., on weekends), thereby facilitating course completion despite irregular participation patterns. Managing missed sessions was thus perceived as unproblematic. Participants also described individual strategies to support engagement, such as preparing necessary equipment in advance and integrating the course into daily routines (e.g., via to-do lists or calendar entries). See Table 3 for corresponding subcategories and respective anchor quotes.

**Table 3:** Perceived challenges and dealing with obstacles: subcategories and anchor quotes

	Subcategory	Anchor quote
Perceived challenges	Maintaining motivation	“I didn’t participate regularly, precisely because it takes place online. So, I’m motivated when the course is on-site, and then I really enjoy doing it, but never at home. So generally speaking, I can’t do much sport at home because I can’t find the motivation. And that’s exactly what happened with the course.” (ID13, Pos. 32)
	Lack of discipline and absence of strict commitment	“Well, because you can somehow ignore everything much more quickly. A fixed date in the week, for example, you have it in your calendar, there’s a certain commitment. Then there’s the contact with people you interact with, which also creates a commitment. So, there are simply different commitments, because you already have something socially, which at some point lead to the fact that you are more likely to do it.” (ID15, Pos. 32)
	Work-related obstacles	“So, I think that’s probably mostly too many activities in other areas. Especially in the office. (…) If you’ve been working too long during the week and don’t have enough time for many other tasks, then it [the course] goes downhill.” (ID12, Pos. 56)
	Scheduling conflicts	“Well, for example, a call comes in or you suddenly have an appointment, for example a doctor’s appointment, so you have to switch [the course] to the afternoon.” (ID07, Pos. 62)
Dealing with obstacles	Consistent attendance due to recorded courses	“But let’s say I made the time at the weekend the latest. If it didn’t work out during the week, I also can finish work at 3 or 4 p.m. when I do home office, so I still have time [for the course]”. (ID02, Pos. 58)
	Successful course completion	“But that wasn’t really a problem with the courses because they weren’t live courses, so I was able to watch them.” (ID01, Pos. 52)
	Organizational strategies	“So, you really must have a structured plan, I have to say. And the appointments that are then included usually work out well.” (ID07, Pos. 72)

### Past and present health behavior

Most participants reported engaging in regular physical activities, such as swimming, basketball and handball, and maintaining a healthy diet during their childhood and early adulthood. In this context, participants described their social environment and families as supportive of an active and health-conscious lifestyle. Conversely, some participants indicated inconsistent health behavior in their past, with minimal or no attention to health-related matters. In these cases, limited or no support from family and social networks during childhood was noted. Many participants assess their current health behavior as positive, emphasizing regular physical exercise and a balanced diet. A few participants reported experiencing health challenges, partly due to life circumstances such as full-time employment, which they perceived as limiting their ability to make optimal health choices. When questioned about the influence of the prevention course on their current health behaviors, most participants acknowledged positive changes. This included increased physical activity, healthier eating habits, improved stress management techniques, and, notably, one participant ceased smoking following course participation.

Overall, participants agreed that their past experiences with health-related issues, particularly within the family environment, significantly shape their current attitudes towards health behaviors, thereby influencing their decision to engage in prevention courses. See Table 4 for corresponding subcategories and respective anchor quotes.

**Table 4:** Past and present health behavior: subcategories and anchor quotes

	Subcategory	Anchor quote
Past health behavior	Positive past health behavior	“I’m really quite grateful to my parents for that. They always made a great effort to keep us kids sporty, that we ate healthily. So that’s what I learned at home, that these are actually the foundations, the building blocks with which you move forward. And above all, that the responsibility for your health primarily lies with yourself and that you don’t say, well, if I’m not feeling well, I’ll just go to the doctor and they have to make sure that I’m well again, but that the doctor is certainly there to support you, but you also have to see what you can do about it yourself.” (ID17, Pos. 93)
	Challenges in past health behavior	“In other words, [my health behavior was] non-existent. It was basically about getting something to eat on the table quickly. I rode my bike to work, but that was usually the only physical activity I did.” (ID09, Pos. 172)
Current health behaviors	Positive current health behavior	“So, when it comes to health behavior, many things play a role, but I think I would describe It [my health behavior was] as a bit above average. I eat quite healthily, I exercise, I’m not an athlete or anything, but I do integrate exercise into my daily routine and I also place a lot of importance on nutrition. (ID11, Pos. 100)
	Challenges in current health behavior	“Unfortunately, I wouldn’t rate it, [my health behavior was] highly, even at this point, because I’m working full-time since the beginning of February. It’s very difficult for me to find the time and motivate myself to do it, because I have so little time. But a month ago, when I still had time, I was much more active and had the motivation. Now, unfortunately it’s not the case, I must work on it. But that’s why, I would describe it more as negative behavior.” (ID13, Pos. 70)
Impact of prevention courses	Positive influence of course participation on present health behavior	I would say definitely better than before I took the courses. Just smoking alone, that’s gone now. […] But I’m really satisfied. I think I’m doing better. I also feel better. So things have definitely changed for the better.” (ID03, Pos. 79)

### Course evaluation and suggestions for future courses

Participants evaluated the courses overall positively, particularly highlighting the role of the course leaders, who were perceived as competent, friendly, and motivating, contributing to an overall positive atmosphere within the courses. The combination of theoretical knowledge and practical exercises was appreciated, as it facilitated the integration of course content into daily life. For example, participants described how courses combined scientific explanations (e.g., on nutrition or smoking-related health effects) with practical elements such as cooking demonstrations or strategies for overcoming cravings (see Supplementary material 1). In addition, participants valued clear course structures (e.g., warm-up, main part, and cool-down in exercise courses in the exercise courses), diverse content, and good technical quality of the video-based format.

However, participants shared both criticism and constructive suggestions. Course levels were sometimes perceived as either too advanced or too basic. As a result, participants expressed the desire for a more extensive range of course levels tailored to both beginners and advanced users, with clear indications of the difficulty level incorporated into the course titles. Beyond that, participants wished for a generally larger selection of courses and more individualized options.

Several suggestions related to the digital format were mentioned. These included continued access to course materials beyond the course period and the provision of follow-up courses that would build upon the content covered in previous courses. Given that the courses were activated only once per week, participants wished for quicker activation as soon as the previous course was completed. Some participants expressed that, in hindsight, they would have preferred to engage in an on-site course. They missed social interactions with fellow members and desired personalized instructions. Finally, they suggested that receiving notifications about the upcoming course content and necessary materials, as well as more reminder emails, would have enhanced their engagement. See Table 5 for corresponding subcategories and respective anchor quotes.

**Table 5:** Course evaluation and suggestions for future courses: subcategories and anchor quotes

	Subcategory	Anchor quote
Positive evaluation	Course leaders	“There were two trainers, and they took turns. But one focused on strength, and the other on endurance. And it was enjoyable, definitely.” (ID09, Pos. 88)
	Course content	“I also found them good because the contents were very diverse, so it was never boring. What I also liked was the theory at the beginning. There was an explanation of the exercises.” (ID05, Pos. 48)
	Well-structured	“Also, the structure, that was all fine, kept quite simple, so to speak. We had the main topic, then it was explained, and individual contributors added their insights. After that, we received a task, and it was all quite manageable, really.” (ID10, Pos. 60)
	Video settings	“I liked, for example, the setting, the background, the recording quality was really good somehow. So, these are all genuinely positive aspects that can be explicitly noted.” (ID15, Pos. 56)
Negative evaluation	Inappropriate course levels	„And maybe for a yoga course, although it was actually for advanced participants, (…), I didn’t find it so challenging.” (ID05, Pos. 78) “(…) that some exercises are a bit challenging for beginners or those who have problems, it was a bit more difficult to perform.” (ID07, Pos. 38)
	Small course selection	“But I find that what is offered to me, it’s just a bit few. Not all courses really fit for me, I would say. They do offer many prevention courses, but I am neither a smoker nor am I heavily overweight. It’s a bit challenging in that regard.” (ID01, Pos. 32)
	Follow-up courses	“So, a follow-up course afterwards would be nice because, as it is, there’s really only that one yoga course and that one Pilates, and there’s nothing like an advanced course or a follow-up course.” (ID01, Pos. 74)

### Results of the on-site course interviews

On-site course attendees mentioned three main differences compared to participants of digital prevention courses. First, recommendations for on-site courses mainly came from their social environment, for instance from friends or peers, whereas digital course participation was more often triggered by institutional communication (e.g., AOK website, newsletter). The second important distinction between the course formats concerns the motivational factor of fostering a sense of community. For participants in the on-site courses, the primary source of motivation stemmed from the shared experience of being together with others and engaging in physical activity as a collective effort, in contrast to digital formats, where social interaction was largely absent and sometimes perceived as a barrier. Finally, on-site courses offered a crucial advantage: the ability for course instructors to provide direct explanations and demonstrations of exercise instructions. This allowed instructors to ensure the correct execution of exercises and make corrections when necessary, which was not possible in digital formats. Moreover, it provided the opportunity to accommodate attendees unable to perform specific tasks due to health reasons (e.g., difficulty getting on their knees) and to offer guidance by demonstrating alternative exercises at an individual level. See Table 6 for corresponding subcategories and respective anchor quotes.

**Table 6:** Subcategories and anchor quotes of the on-site course interviews (contextual comparison with digital formats)

	Subcategory	Anchor quote
Motivating factors	Sense of community	“If you are truly present with people, it creates a completely different atmosphere. For me, it was really about connecting with others, getting to know a few people, laughing together, chatting away. It’s a whole different experience compared to being on the computer, where everyone sits quietly, doing their exercises. Being in an actual group, having a good time together, that’s something else.” (ID21, Pos. 48).
	Direct feedback of instructors	“And the course instructor ensures that you’re doing the exercises correctly. They also take into consideration if you have any complaints, adjusting the approach and suggesting alternatives.” (ID23, Pos. 46).

**Fig. 2:**
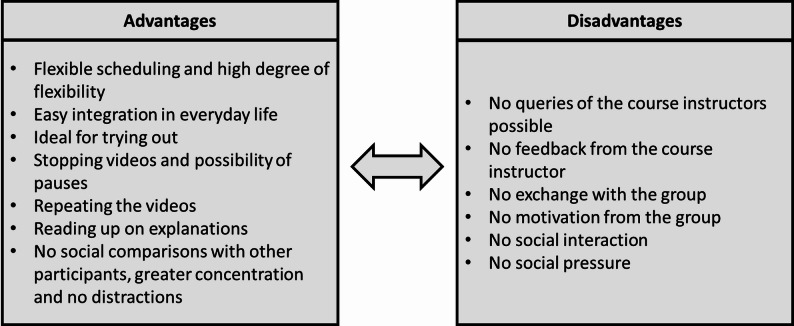
Advantages and disadvantages of digital prevention courses

An overview of the advantages and disadvantages of digital compared to on-site prevention courses, as reported by both digital and on-site course participants, is summarized in Fig. 2.

## Discussion

This study explored motivating factors and barriers to participation in digital prevention courses offered by two statutory health insurance funds in Germany and included a contextual comparison with on-site formats. Digital prevention courses were generally perceived positively, with participants highlighting their accessibility and flexibility. At the same time, several structural and individual barriers, as well as format-specific disadvantages, were identified that warrant further consideration.

While many of the identified factors have been described in previous research, this study adds to existing literature in several important ways. First, it provides an integrated perspective on motivating factors, perceived barriers, and structural course characteristics, thereby offering a more comprehensive understanding of participation in digital prevention programs. Second, by embedding the analysis within the specific context of the German statutory health insurance system, the study contributes context-sensitive insights into how digital prevention services are used in a healthcare system with comparatively broad access to conventional services. Third, the inclusion of a comparative perspective between digital and on-site formats allows for the identification of key trade-offs between flexibility and social interaction. Finally, the findings contribute to the identification of general design considerations for digital prevention programs, highlighting the importance of combining flexible access with structured support and opportunities for continued engagement.

The most prominent drivers of participation were temporal flexibility and the convenience of accessing courses from home, findings consistent with prior research highlighting flexibility as a key advantage of digital prevention formats [22, 34]. These low-threshold characteristics appear particularly relevant for participants with family obligations or irregular working hours. In addition, participants pursued specific health-related goals and described positive changes in their behaviors, including healthier dietary habits, increased physical activity, and improved stress management. While these findings suggest that digital prevention courses can support individual health goals, it remains unclear to what extent such changes can be sustained over time [6].

Despite these benefits, several barriers were identified. Participants reported difficulties with self-discipline, a lack of social interaction, and problems integrating courses into their daily routines. These barriers resonate with earlier studies showing that digital health programs place higher demands on individual self-regulation and fail to reproduce the group dynamics of on-site formats [22, 34, 35]. This highlights that flexibility alone may not be sufficient to ensure sustained participation [22]. At the same time, the availability of recorded sessions was perceived as a major strength, enabling participants to revisit content asynchronously and to compensate for missed sessions, thereby supporting course completion.

The contextual comparison with on-site course formats highlighted distinct differences in participation pathways and motivational dynamics. Digital participants often became aware of courses through institutional communication, whereas on-site participation was frequently encouraged by social networks. On-site attendees particularly valued the sense of community and mutual motivation derived from exercising together, as well as the ability of instructors to provide direct feedback and adapt exercises to individual needs. These findings illustrate a trade-off: while digital formats maximize flexibility and reach, on-site courses offer social support and personalized guidance. Building on these findings and the relationships illustrated in Fig. 2, hybrid approaches that combine digital flexibility with elements of interpersonal interaction, such as feedback from instructors or structured group components, may be particularly promising. Integrating digital prevention courses with existing on-site services could help to leverage the strengths of both formats and address key barriers identified in this study [35]. In the German context, such hybrid or complementary formats are already supported by statutory health insurance funds under the legal framework of Social Code Book V (§ 20 SGB V) [5]. Future research should therefore more explicitly examine participation behaviors in hybrid prevention models to identify effective strategies for sustainable prevention.

In the present study, participants evaluated instructors and course structure positively. Course leaders were perceived as competent and motivating, and course content was engaging and informative. However, participants also raised critical points. Specifically, variation in course levels and limited opportunities for follow-up courses indicate the need for more differentiated and continuous program structures. Taken together, these findings point to general design considerations for digital prevention programs beyond the specific context of the AOK courses. They highlight the importance of combining structured yet flexible content delivery with clear differentiation of course levels and opportunities for continued engagement. These results further suggest that digital prevention programs should not be viewed as isolated interventions but rather as part of a continuous preventive pathway that extends beyond single course participation. In this context, different stakeholders across the healthcare system need to collaborate to develop coherent structures that connect preventive offers with broader health promotion strategies and long-term support [1, 12, 14].

Beyond these individual experiences, participation in prevention programs is shaped by broader demographic and socioeconomic factors. Previous research has shown that factors, including age, gender, and educational level, strongly influence the likelihood of engaging in preventive measures [1, 11]. Digital formats may lower certain barriers, yet they simultaneously introduce new prerequisites. Against this background, health literacy, and particularly digital health literacy, emerge as a critical determinant of access. As information, registration, and communication about preventive offers increasingly take place online, individuals require adequate skills to find, evaluate, and use digital health offers effectively [14, 26, 28, 35]. Without these competencies, vulnerable groups risk being excluded, reflecting the inequality paradox of prevention, whereby prevention programs tend to benefit primarily those who are already health-conscious and socioeconomically advantaged [1, 36, 37]. Strengthening health literacy in general, and digital health literacy in particular, is therefore essential to ensure that preventive pathways remain accessible and socially equitable.

Beyond issues of access and equity, the sustainability of behavioral changes achieved through digital prevention programs also requires further investigation. While participants in the present study reported positive short-term effects on their health behaviors, evidence regarding the long-term maintenance of such changes remains limited [6, 22]. In addition, the relatively high degree of self-regulation required in digital formats, as well as the limited social interaction, may pose challenges for sustained behavior change over time. Future research should therefore examine the mid- and long-term impacts of digital prevention courses and systematically compare different course formats, such as digital, on-site, or hybrid approaches, regarding their ability to support sustained behavioral change. From a practical perspective, strategies to enhance long-term engagement may include structured follow-up offers, opportunities for reflection and goal setting, and the integration of human support elements, such as feedback from health professionals or guided check-ins. Such approaches may help to complement digital formats and strengthen their potential to support lasting behavior change.

### Limitations and future directions

The findings of this study need to be interpreted considering certain limitations. The sample was small and restricted to insured individuals of two regional statutory health insurance funds, which limits the generalizability of the results. Participation was voluntary and may have attracted individuals with a pre-existing interest in health promotion, potentially introducing self-selection bias. To gain a more comprehensive understanding of motivational factors for participation in digital prevention courses, future studies should therefore include more diverse groups that are typically underrepresented in such programs. In particular, only one male participant was included in the present study, most participants had a high educational background, and older adults were underrepresented. This constitutes a key limitation, as these groups may differ in their motivations, barriers, and preferences regarding digital prevention programs.

Another limitation relates to the assessment of individual characteristics that may contribute to participation in digital prevention courses. While participants’ past and present health behaviors were explored qualitatively, more detailed information on objective health status (e.g., specific medical conditions or severity of illness) was not systematically assessed. Therefore, no conclusions can be drawn regarding how health status may relate to participation. In addition, digital competence and access to digital infrastructure were not explicitly examined, although both factors are likely to shape individuals’ ability and willingness to engage in digital prevention programs.

To strengthen the overall results, future studies should involve larger and more diverse samples and validate the findings in large-scale quantitative surveys, enabling a more systematic analysis of associations between individual characteristics and participation behavior. Furthermore, the study does not allow conclusions about long-term behavioral outcomes, highlighting the need for longitudinal research on the long-term effects of digital prevention courses on health behavior. Despite these limitations, the study provides valuable insights into user perspectives and offers important implications for the further development of digital prevention programs.

## Conclusion

Taken together, our findings suggest that digital prevention courses can play an important role in expanding access to preventive health programs, however, their potential to promote equitable access remains limited if they primarily reach individuals who are already socioeconomically advantaged and health conscious. In line with our sample characteristics, participation appears to be shaped by existing individual and structural resources, which may reinforce rather than reduce inequalities in prevention. To address these challenges, digital programs should be further embedded in continuous preventive pathways and complemented by targeted strategies that actively engage underserved populations, such as tailored outreach, support for digital health literacy, and the integration of interpersonal elements. Hybrid approaches that combine digital flexibility with human support may help to reduce barriers and strengthen sustained participation. Future developments in this field will need to carefully balance flexibility, accessibility, and social connectedness to maximize both reach and effectiveness.

## Supplementary Information

Supplementary Material 1.

Supplementary Material 2.

Supplementary Material 3.

Supplementary Material 4.

Supplementary Material 5.

## Data Availability

The interview guide and coding framework are provided in Appendix A and Appendix B. The original German interviews are not publicly available, as participants did not provide consent for data sharing. For language editing and readability, the authors used ChatGPT (version 5) during the preparation of this manuscript. All content was subsequently reviewed and revised by the authors, who take full responsibility for the final version of the publication.

## Abbreviations

AOK: Allgemeine Ortskrankenkasse (General local health insurance fund)
COREQ: Consolidated Criteria for Reporting Qualitative Research
DiPaH: Digital health preventive measures for arterial hyptertension
G-BA: Gemeinsamer Bundesausschuss (Federal Joint Committee)
GKV: Spitzenverband Bund der Krankenkassen (National association of staturatory health insurance funds)
HLS-GER: Health Literacy Survey Germany
SGB V: Sozialgesetzbuch V (Social Code Book V)
ZPP: Zentrale Prüfstelle Prävention (Central Prevention Testing Center)
