# Supplementary material for: Motivating factors and possible barriers to participation in digital prevention courses of two statutory health insurance funds in Germany: a qualitative interview study
Source: BMC Public Health. 2026 Jul 7;26:2071. doi: 10.1186/s12889-026-28392-z (PMC13343681; doi:10.1186/s12889-026-28392-z)
Supplement: Supplementary file 1 — Supplementary Material 1. [file 12889_2026_28392_MOESM1_ESM.docx]

Appendix B. Coding framework.

| **Category** | **Subcategory** | **Coding rule** |
| --- | --- | --- |
| Motivating and supporting factors for course participation | Flexible scheduling and a high degree of flexibility | Coded when participants describe time flexibility, convenience, or low preparation effort. |
|  | Achievement of health goals | References to participants’ personal goals (e.g., fitness, diet, stress reduction, smoking cessation). |
|  | Free access | Mentions of cost-free participation as a motivator. |
|  | Presentation of content and increase in knowledge | Statements about informative or well-presented content and knowledge gain. |
|  | Multiple viewing and repetition of exercises | Mentions of reminder emails or the ability to rewatch content. |
| Perceived challenges and dealing with obstacles | Maintaining motivation | References to participants’ challenges in maintaining motivation at home. |
|  | Lack of discipline and absence of strict commitment | Descriptions of participants’ reduced engagement due to missing structure or commitment. |
|  | Work-related obstacles and scheduling conflict | Passages on work, family, or scheduling conflicts interfering with participation. |
|  | Consistent attendance due to recorded courses | Statements about the use of recorded sessions. |
|  | Successful course completion | Mentions of full course completion. |
|  | Organizational strategies | References to organizational strategies (e.g., scheduling, preparing materials). |
| Past and present health behavior | Positive past health behavior | Descriptions of a physically active or health-conscious lifestyle in childhood and early adulthood. |
|  | Challenges in past health behavior | Statements on poor health-related behavior in childhood and early adulthood. |
|  | Positive current health behavior | Reports on positive present health behavior (e.g., physical exercise, healthy diet, and stress management). |
|  | Challenges in current health behavior | Statements on poor present health behavior. |
|  | Positive influence of course participation on present health behavior | Descriptions of participants’ positive behavioral changes attributed to course attendance. |
| Course evaluation and suggestions for future courses | Course leaders | Comments on the competent and motivating instructors and their diverse areas of expertise. |
|  | Course content | Passages on the diverse and high-quality course contents. |
|  | Well-structured | Mentions on the well-structured and organized courses. |
|  | Video settings | Descriptions of the video setting. |
|  | Inappropriate course levels | Statements that course content was too basic or too advanced. |
|  | Small course selection | Mentions of missing or insufficient course topics. |
|  | Follow-up courses | Requests for continued access, advanced levels, or follow-up offers. |
| Contextual on-site comparison | Social network recruitment | References to participation encouraged by friends, family, or peers. |
|  | Community and motivation | Emphasis on the shared experience and mutual encouragement. |
|  | Instructor feedback | Mentions of individualized corrections and adaptations by instructors. |
