# Supplementary material for: Motivating factors and possible barriers to participation in digital prevention courses of two statutory health insurance funds in Germany: a qualitative interview study
Source: BMC Public Health. 2026 Jul 7;26:2071. doi: 10.1186/s12889-026-28392-z (PMC13343681; doi:10.1186/s12889-026-28392-z)
Supplement: Supplementary file 2 — Supplementary Material 2. [file 12889_2026_28392_MOESM2_ESM.docx]

Appendix A. Interview guide (translated from German)

| **Intro** |  |
| --- | --- |
| **Formalities and health goals** | - You participated in a prevention course offered by AOK. Could you tell me about it? - What was the topic of the course (e.g., exercise, nutrition, relaxation)? - Was it a CyberFitness course? - When did you take part? - Which format was it (on-site or digital)? - What motivated you to choose this specific course and topic? - Did you pursue any health goals? - How did you learn about the course / how did you register? |
| **Motivation and facilitating factors** | It is one thing to start a course, but another to stay engaged over time.   - What motivated you to take part in the course? - What helped you stay motivated from week to week? - Were there any factors that supported your participation? - Were there people who encouraged or supported you? - How did it help that the course was conducted online? - What advantages do you see in online courses compared to on-site ones? - And what disadvantages? Could you elaborate on that or give an example? |
| **Barriers** | Sometimes things do not go as planned.   - Were there occasions when you missed a session? - What were the reasons for this? - Were you able to overcome these challenges? - How often did this occur, and how did you manage it? - Were there other factors that interfered with participation? |
| **Evaluation** | - How did you like the course overall? - To what extent were your expectations met? - What benefits did you take away from the course? - What would you have wished for ideally? - Could you elaborate or give an example? |
| **Health behavior** | - How would you describe your current health behavior? - What is your general attitude toward health and physical activity? - Do you think the course influenced your health behavior? In what way? - How would you describe your past health behavior? - What role have health and vitality played in your life? - Do you think your attitude toward health influences your use of preventive programs? If so, how? |
| **Biographical influences** | - Were there periods in your life when you lived particularly health-consciously? What did that look like? - Thinking back to your childhood and adolescence, what role did health and vitality play for you? - How was health perceived in your family or social environment? - In your view, how does your earlier health behavior influence your current use of prevention programs? Could you give an example? |
| **Feedback** | - Is there anything you would like to add? - Do you have any general feedback on the interview, the online questionnaire, or the prevention course? |
| **Sociodemographic information** | Finally, I would like to ask a few short questions about you personally:   - Age - Gender - Place of residence - Educational background (highest degree obtained) - Occupation - Hypertension diagnosis or risk |
| **Closing remarks** |  |
