# Supplementary material for: Motivating factors and possible barriers to participation in digital prevention courses of two statutory health insurance funds in Germany: a qualitative interview study
Source: BMC Public Health. 2026 Jul 7;26:2071. doi: 10.1186/s12889-026-28392-z (PMC13343681; doi:10.1186/s12889-026-28392-z)
Supplement: Supplementary file 3 — Supplementary Material 3. [file 12889_2026_28392_MOESM3_ESM.docx]

Supplementary material 1. Reported structure and content of digital prevention courses based on participant interviews.

| **Course type** | **Core topics** | **Theoretical components (reported)** | **Practical components (reported)** | **Illustrative examples from participant reports** |
| --- | --- | --- | --- | --- |
| Nutrition | Healthy eating, weight management | Nutritional knowledge, psychological aspects of eating behavior | Cooking demonstrations, meal planning | Participants reported receiving practical recipes and guidance on everyday food choices, as well as insights into psychological aspects of eating behavior. |
| Physical activity (e.g. back fitness, yoga) | Strength, endurance, mobility | Exercise principles, body awareness | Guided exercises (warm-up, main part, cool-down) | Participants described structured sessions including warm-up, main exercises, and cool-down, sometimes with different instructors focusing on strength and endurance components. |
| Stress management | Stress reduction, coping strategies | Background on stress and its effects | Relaxation techniques, practical exercises | Participants reported that courses covered a range of topics including work-related stress, lifestyle factors, and coping strategies. |
| Smoking cessation | Addiction mechanisms, health effects | Physiological and psychological aspects of smoking | Strategies to manage cravings | Participants described learning about the health effects of smoking and receiving practical strategies to cope with cravings. |
