# Supplementary material for: Motivating factors and possible barriers to participation in digital prevention courses of two statutory health insurance funds in Germany: a qualitative interview study
Source: BMC Public Health. 2026 Jul 7;26:2071. doi: 10.1186/s12889-026-28392-z (PMC13343681; doi:10.1186/s12889-026-28392-z)
Supplement: Supplementary file 4 — Supplementary Material 4. [file 12889_2026_28392_MOESM4_ESM.docx]

Supplementary material 3. Additional information to COREQ checklist.

| Characteristic | Description |
| --- | --- |
| Credentials | MB, TT, FM, GF, SM and ML hold doctoral degrees. DB and FS hold master’s degrees. SSp holds a professorship. |
| Occupation | MB, TT, GF, and ML are working as consultants at revFLect GmbH with a focus on health care.  FM, FS and SM work at Brandenburg Medical School (MHB) and DHZC Charité, focusing on health care research.  DB is a health services researcher at MHB and a clinical psychologist at the Immanuel Klinikum Bernau Heart Center Brandenburg.  SSp works at DHZC Charite, focusing on health care research. |
| Gender | MB, TT, DB, SM, and FS are female. FM, SSp, GF, and ML are male. |
| Experience and training | MB, TT, DB, SM, FM, FS, SSp, ML, and GF have backgrounds in psychology, health sciences, or related life sciences; SSp is additionally trained as a cardiologist. All authors have experience in health research and have co-authored multiple scientific publications. Several members of the research team (DB, SM, FM, FS, and SSp) have prior experience in qualitative research, including conducting and analyzing interviews. |
